# A review of the current evidence for maintenance therapy in gastric cancer

**DOI:** 10.3389/fphar.2026.1667453

**Published:** 2026-03-17

**Authors:** Xinpu Han, Yu Zhang, Qiuyue Fan, Jiahui Yu, Liyuan Lv, Ya Li, Li Hou

**Affiliations:** 1 Department of Oncology and Hematology, Dongzhimen Hospital, Beijing University of Chinese Medicine, Beijing, China; 2 Beijing University of Chinese Medicine, Beijing, China; 3 Peking University Cancer Hospital, Beijing, China

**Keywords:** biomarkers, clinical applications, gastric cancer, maintenance therapy, mechanism of action

## Abstract

**Objectives:**

Gastric cancer (GC) is usually diagnosed at an advanced stage, and although partial or complete remission can be achieved after first- or second-line treatment, minimal residual disease may remain, with the potential risk of repopulation and recurrence. The main goals of maintenance therapy (MT) at this stage are to prolong progression-free survival (PFS) and overall survival (OS), attenuate adverse events (AEs), and maintain quality of life (QoL). In recent years, there has been a gradual increase in studies on maintenance therapy in advanced and metastatic GC. In this article, we systematically review the studies on MT in GC to assess the current knowledge on the mechanism of action, clinical applications, and biomarkers of this treatment approach.

**Methods:**

We searched Embase, Web of Science, PubMed, and Cochrane Library databases, including the period from the inception of the databases through 6 June 2025. Searches were conducted using search terms related to GC and MT. The primary outcomes were PFS and OS, while secondary outcomes included AEs and QoL.

**Results:**

The core mechanism of MT is to inhibit the proliferation and recurrence of tumor cells through continuous low-intensity treatment. Specific mechanisms include inhibiting angiogenesis and tumor cell proliferation, regulating the tumor microenvironment (TME), enhancing the body’s immune surveillance and clearance of tumors, and regulating tumor dormancy. In clinical practice, sustained low-dose application of single chemotherapeutic agents, targeted agents, immune checkpoint inhibitors, and combinations as the mainstay of MT can be clinically important in the maintenance phase of GC patients by inhibiting tumor growth, proliferation, and recurrence to prolong the PFS and OS, while improving QoL. Among them, capecitabine, S-1, bevacizumab, and avelumab were most frequently evaluated. Biomarkers are crucial for predicting treatment response and efficacy in GC MT, monitoring treatment effectiveness, assessing prognosis, and optimizing drug development. Hemoglobin levels, programmed cell death ligand 1 combined positive score, immune (biomarker-positive) or angiogenesis-dominant (biomarker-negative) status, TME characteristics, and C-X-C motif chemokine ligand 12 have shown potential use as indicators for assessing the efficacy of GC MT.

**Conclusion:**

MT, whether applied as a continuous or switching strategy, may sustain clinical benefits without compromising QoL due to severe AEs. Future studies should investigate the long-term clinical benefits of MT and its impact on resource utilization and health-related QoL.

## 1 Introduction

The incidence of gastric cancer (GC) ranks fifth among global cancer cases, and its mortality rate is even higher, ranking fourth. Both the incidence rate and mortality rate continue to increase. According to data from the 2021 Global Burden of Disease Study, there were more than 1.23 million incidence cases and approximately 950,000 deaths, imposing a significant burden on public health (Qin et al., 2025). At present, the main treatment methods for GC include surgical treatment, chemotherapy, radiotherapy, targeted therapy, and immunotherapy. In advanced gastric cancer (AGC), first-line (1L) combination chemotherapy, targeted therapy, and monoclonal antibodies, such as trastuzumab in HER2-positive GC, bevacizumab combined with chemotherapy, and second-line treatments such as taxane, irinotecan, and ramucirumab have been proven to improve survival rates (Song et al., 2016; Ohtsu et al., 2011; Kanagavel et al., 2015). A meta-analysis also indicated that the therapeutic effect of GC patients receiving second-line chemotherapy with irinotecan or docetaxel was significantly better than that of the best supportive care (Kim et al., 2013). However, most GC patients are already at an advanced stage of the disease at the time of diagnosis. After the initial standard treatment, there are still problems such as a high recurrence rate and poor tolerance of some patients to subsequent treatments, resulting in a poor prognosis. The 5-year survival rate of AGC patients is less than 5%, which leads to the inability of patients to benefit in the long term. This has promoted the continuous optimization and innovation of GC treatment strategies (Siegel et al., 2024). In recent years, with the progress in fields such as chemotherapy, targeted therapy, and immunotherapy, the treatment mode of GC has gradually shifted from single chemotherapy to multimodal comprehensive treatment. Among these, maintenance therapy (MT), an important treatment strategy, has received extensive attention.

MT refers to a treatment strategy in which cancer patients continue to use lower doses or different types of drugs after completing their initial treatments, aiming to control disease progression, prolong progression-free survival (PFS) and overall survival (OS), reduce toxicity, and improve quality of life (QoL) by balancing efficacy and toxicity (Berinstein, 2006). MT often uses PFS, OS, and security as the main evaluation indicators. Therefore, maintaining the therapeutic effect while reducing toxic reactions after the initial treatment has become an important research direction in the treatment of GC. It is essential to clarify that, although MT and adjuvant therapy are both important components of comprehensive cancer treatment and aim to prevent recurrence, they differ significantly in terms of treatment timing, core objectives, target populations, and strategies. Adjuvant therapy is initiated after radical treatment for operable early or locally advanced AGC (stages I–III), aiming to “clear microscopic residual lesions and cure the disease.” The treatment course is fixed and often involves potent chemotherapy or combination regimens [such as SOX (oxaliplatin + tegafur–gimeracil–oteracil potassium) and XELOX (oxaliplatin + capecitabine)] (Kilic et al., 2016; Kanda et al., 2015). For AGC/metastatic gastric cancer (MGC) (stage IV), MT is administered after achieving disease control in the first/second line of treatment. Its focus is on “long-term control of the disease, prolongation of survival, and maintenance of QoL.” It uses drugs with low toxicity and good tolerance (such as capecitabine and tegafur–gimeracil–oteracil potassium [S-1]) for long-term treatment until disease progression occurs. The core concept is “living with the disease” (Berinstein, 2006). MT and adjuvant therapy have a complementary relationship. In some cases, they can be indirectly connected. For example, in the case of locally AGC where adjuvant therapy fails and the disease progresses to advanced stage, if 1L treatment is effective, it can be transferred to MT. However, there is no direct substitution relationship. Clearly defining the boundaries between MT and adjuvant therapy can provide a clear basis for clinical decision-making. At present, MT has become part of the standard treatment regimens for various cancers, such as GC, colorectal cancer, lung cancer, breast cancer, and ovarian cancer (Song et al., 2016; Foster et al., 2009; Paz-Ares et al., 2013; Roviello et al., 2021; Yalcin et al., 2013; Wang et al., 2021). With the rapid increase in research on MT over the past 5 years, significant progress has been made in elucidating the mechanisms of action and potential therapies for GC maintenance. Therefore, we conduct a systematic review of the recent literature related to the MT of GC. The purpose of this review is to evaluate the mechanisms of action of MT, its current use, clinical applications, patient outcomes with and without MT, and future research directions in this field.

## 2 Drugs for MT and their mechanisms of action

**FIGURE 1:**
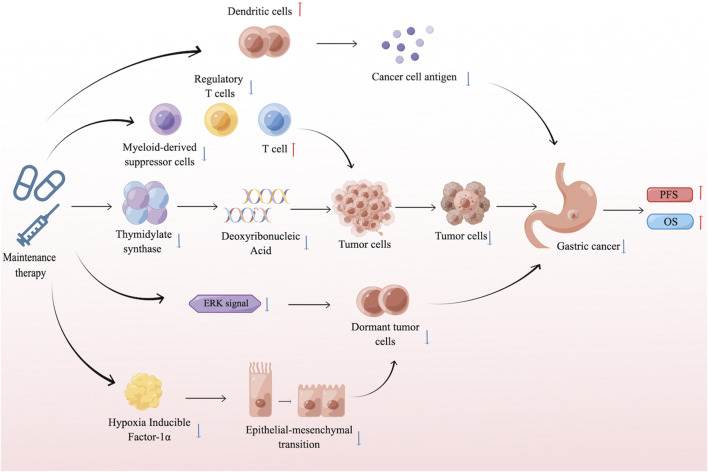
Mechanism of maintenance therapy for gastric cancer.

The core mechanism of MT lies in inhibiting the proliferation and recurrence of tumor cells through continuous low-intensity treatment. The biological effects of MT depend on the specific drug type. Different drugs exert their functions by inhibiting angiogenesis and tumor cell proliferation, regulating the tumor microenvironment (TME), enhancing the body’s immune surveillance and tumor clearance, and modulating tumor dormancy (Figure 1). Understanding the mechanisms of action in MT of GC is of great significance for further optimizing treatment plans and improving patient prognosis.

### 2.1 Chemotherapeutic drugs

Chemotherapeutic drugs are the traditional choice for MT of GC. Low-dose chemotherapy (LDM) is a commonly used maintenance regimen for GC. Studies have found that the maximum tolerated dose (MTD) of chemotherapy and LDM have opposite effects on the mobilization and survival ability of circulating endothelial progenitor cells. LDM hinders DNA replication and cell division in cancer cells by continuously inhibiting thymidylate synthase and DNA synthesis pathways. It is more effective than MTD in targeting cells involved in tumor angiogenesis, thereby reducing the likelihood of tumor regeneration (Untergasser et al., 2006). LDM docetaxel has been found to have anti-angiogenic activity in both *in vitro* and *in vivo* studies, which can affect microvascular growth and has no toxic effect on nude mice (Wu et al., 2011). The beneficial effect of cyclophosphamide (CTX) in enhancing anti-tumor immunity may be due to its ability to eliminate regulatory T cells (Tregs), reset the homeostasis of dendritic cells (DCs) in the tumor host, and restore the homeostasis-driven expansion of the original tumor-specific effector T cells (Ghiringhelli et al., 2004). Studies have found that long-term administration of low-dose CTX can induce beneficial immune regulation, prevent the renewal of Tregs in tumor-bearing mice, and restore an effective immune response to tumor cells, thereby prolonging survival time and preventing disease recurrence (Liu et al., 2007; Lutsiak et al., 2005; Taieb et al., 2006). Veltman et al. (2010) found that continuous LDM administration is beneficial for breaking immune tolerance by eliminating Tregs, and its regulatory effect on DC-based immunotherapy can lead to an increase in the survival rate of tumor-carrying mice. It should be noted that high paclitaxel (PTX) concentrations induce apoptosis of DCs, while low concentrations not only reduce the apoptosis of these cells but also prevent the inhibitory effect of tumors on DC maturation, induce the phagocytosis of tumor antigens by DCs, and enhance the induction of their maturation and anti-tumor immune responses (He et al., 2011). Lin et al. (2022) found that low-dose PTX increased the expression level of programmed death-ligand 1 (PD-L1) and the number of CD8^+^ T cells in GC mice, while low-dose 5-fluorouracil (5-FU) reduced the number of bone marrow-derived suppressor cells and programmed death-1 (PD-1)^+^ CD8^+^ T cells. Wang et al. (2013) also found that the combination of wogonin and low-dose PTX can promote the apoptosis of GC cells and inhibit tumor growth, while the combined application of wogonin and high-dose PTX may produce antagonistic effects. LDM drugs (such as CTX) can selectively deplete Tregs and restore the anti-tumor activity of CD8^+^ T cells.

### 2.2 Targeted drugs

Targeted drugs, by acting on tumor-specific molecular targets, have become one of the important treatment options for MT of GC. The mechanism focuses on anti-angiogenesis and the regulation of tumor dormancy. Anti-angiogenesis is one of the core strategies in MT of GC. By targeting the vascular endothelial growth factor (VEGF) pathway, it blocks the formation of new blood vessels in tumors, cuts off the supply of nutrients and oxygen to cancer cells, and simultaneously inhibits the proliferation of microscopic residual lesions and vascular infiltration, thereby delaying the progression of the disease. Ramucirumab and bevacizumab targeting the vascular endothelial growth factor receptor (VEGFR) can reduce blood supply to GC cells, leading to hypoxia and nutrient deficiency in cancer cells, ultimately reducing their proliferation ability and prolonging OS (Guan et al., 2023; Wöll et al., 2017). In animal models, it was observed that combining anti-tumor drugs with anti-angiogenic agents, such as anti-VEGF, β-cyclodextrin tetradecyl sulfate, tetrahydrocortisol, and endocrine hormones, significantly increased the survival of tumor-bearing animals (Bello et al., 2001; Klement et al., 2002; Soffer et al., 2001; Teicher et al., 1992).

Tumor dormancy is an important target for MT of GC. During tumor treatment, approximately 0.1% of cancer cells do not die directly under the stress of treatments such as radiotherapy and chemotherapy. Instead, cell cycle regulation is triggered to induce dormancy to evade killing and form dormant tumor cells (DTCs). Dormancy may arise from a single cell remaining in a long-term dormant state or from small populations of cells existing in a balanced state as micrometastasis, resulting in no net change in tumor size (Townson and Chambers, 2006). DTCs refer to tumor cells with a stagnant cell cycle and therapeutic tolerance (Phan and Croucher, 2020). At this time, cancer cells exhibit slow division, extremely low metabolic activity, inhibited autophagy, and reduced responsiveness to external stress. Unlike the proliferation pattern of normal cells, DTCs possess their own cellular rhythms. They can undergo cycles of dormancy–activation–subclinical growth until metastasis and recurrence occur under the influence of external stress (Phan and Croucher, 2020). MT mainly inhibits the reactivation of DTCs by regulating cell cycle arrest and microenvironment dependence. Studies have shown that DTCs usually rely on signaling pathways, such as NR2F1 and p38MAPK, to maintain a quiescent state. NR2F1 can promote dormancy by inhibiting MYC and genes related to cell proliferation, while p38MAPK can maintain cells in the G0/G1 phase by antagonizing ERK signaling (Epstein, 2005). MT can inhibit ERK signals through targeted therapy and promote the maintenance of DTCs in a dormant state. Furthermore, changes in the TME (such as hypoxia, immune response, and angiogenesis disorders) can maintain the persistent existence of the DTC state (Balayan and Guddati, 2022). Low-dose anti-angiogenic drugs (such as apatinib) can maintain a moderate hypoxic state and reduce hypoxia-inducible factor-1α (HIF-1α)-driven epithelial–mesenchymal transition and invasion and metastasis.

### 2.3 Immunosuppressants

In GC, the TME usually exhibits an immunosuppressive state, characterized by increased Tregs and myeloid-derived suppressor cells (MDSCs) and high PD-L1 expression; this environment impairs the functions of cytotoxic T cells and natural killer (NK) cells, promotes immune escape, and significantly influences GC progression and treatment resistance (Zou and Restifo, 2010). The core mechanism of immunotherapy in MT for GC lies in reshaping the body’s anti-tumor immune response and regulating the TME, thereby achieving continuous elimination and long-term control of residual cancer cells (Michaud et al., 2009). Its effects can be mainly attributed to three aspects.

First, by continuously blocking the immune checkpoint pathways such as PD-1/PD-L1 or cytotoxic T lymphocyte-associated antigen 4 (for example, through the application of drugs such as nivolumab and pembrolizumab), competitive binding is achieved between the immune checkpoint molecules on GC cells and the surface of T cells, restoring the recognition and killing activity of T cells and GC cells, reversing the inhibitory state of T-cell function, breaking the immune escape mechanism of tumors, and reconstructing the immune surveillance function. Second, this strategy enhances antitumor immunity by dynamically adjusting the immunosuppressive components in the TME, including reducing the infiltration of Tregs and MDSCs, promoting the polarization of M1-type macrophages, decreasing the proportion of inhibitory cells such as M2-type macrophages and the secretion of transforming growth factor-β and other immunosuppressive factors, promoting the infiltration of effector T cells and NK cells and the secretion of pro-inflammatory cytokines such as interferon-γ and interleukin-2, transforming “cold tumors” into “hot tumors,” strengthening the local anti-tumor immune response, and thereby maintaining a persistent anti-tumor immune response. Furthermore, the activated anti-tumor immune network can precisely eliminate residual microscopic lesions, circulating tumor cells, and DTCs after 1L, thereby preventing recurrence and metastasis at the source. It also induces specific T-cell clonal expansion and the formation of immune memory. Even after treatment discontinuation, this network can retain the ability to recognize tumor neoantigens and rapidly initiate an immune response upon cancer cells reappearance, providing long-term immune protection and ultimately prolonging disease control and patient survival.

### 2.4 Mechanistic basis of long-term MT

For patients with AGC, even if partial or complete remission is achieved after 1L treatment, minimal residual disease may still exist. Even when the tumor diameter is ≤ 5 mm, before it can be clinically diagnosed, the tumor cells may have already disseminated from the primary site, serving as “seeds” for long-term recurrence and metastasis (Bouferraa et al., 2022). Therefore, these residual cells have the potential risk of re-proliferation and recurrence. From the perspective of treatment strategies, the MT for GC can serve as the core mechanism of a long-term treatment plan because it achieves a dynamic balance between tumor control and patient tolerance through a dual approach of “continuous targeted intervention + optimization of low-toxicity regimens.” On the one hand, MT blocks the key link of tumor recurrence through “continuous drug pressure”: the residual microscopic lesions, circulating tumor cells, and DTCs remaining after the initial treatment, although they have not yet shown obvious progression, possess the potential for proliferation and recovery. MT, through long-term low-intensity drug intervention, can continuously inhibit DNA replication, angiogenesis, and abnormal activation of tumor cell signaling pathways, preventing residual cells from re-entering the proliferation cycle after drug withdrawal and thereby reducing the risk of recurrence at its source. This continuous intervention does not simply maintain the initial treatment intensity. Instead, it precisely targets the biological characteristics of residual lesions, specifically focusing on key processes such as tumor proliferation and dormancy regulation, thereby achieving the long-term disease management goal of “controlling rather than killing.” On the other hand, MT reduces the accumulation of toxicity during long-term treatment through strategic selection of the medication regimen. In terms of drug types, low-toxicity single drugs or mild combination regimens should be given priority. It can simultaneously inhibit cell proliferation and angiogenesis, thereby reducing the risk of drug resistance, avoiding the toxic superposition of high-intensity combined chemotherapy, and reducing the long-term accumulation of serious adverse reactions such as bone marrow suppression and digestive tract reactions. In terms of dose design, a low-dose and long-cycle administration mode is adopted, which not only maintains a stable blood drug concentration to continuously inhibit tumors but also avoids tissue damage caused by the maximum tolerated dose, thereby enhancing patients’ long-term medication compliance. In terms of protocol adjustment, by adopting the “continuous treatment” or “switching treatment” strategy, the expansion of drug-resistant clones caused by long-term use of a single drug can be prevented. For instance, after 1L is effective, it can be switched to targeted or immune drugs for maintenance. Through the alternations of drugs with different mechanisms of action, the occurrence and accumulation of drug-resistant mutations can be delayed. This strategic design enables MT to maintain continuous tumor control during long-term treatment while minimizing treatment-related damage to the greatest extent, providing core support for AGC patients to achieve the long-term goal of “living with the disease.”

## 3 Treatment strategy

### 3.1 Current status of guideline recommendations

Current clinical guidelines—including the National Comprehensive Cancer Network (NCCN) clinical practice guidelines, the European Society for Medical Oncology (ESMO) clinical practice guidelines, and the Chinese Society of Clinical Oncology (CSCO) guidelines—recommend that patients with GC receive MT with less toxic 5-FU class chemotherapy drugs (capecitabine or S-1) to reduce toxicity associated with prolonged intensive combination regimens (Ajani et al., 2022; Lordick et al., 2022; Wang et al., 2024a).

Several studies have explored the efficacy and safety of capecitabine as a monotherapy in the MT of AGC and MGC. The results showed that it had a positive effect on prolonging the PFS and OS of patients, and the treatment-related adverse events (TRAEs) were controllable. Eren et al. (2016) reported the experience of using capecitabine as a maintenance agent for patients with AGC. The results showed that no treatment-related deaths were observed due to the use of capecitabine. The median progression-free survival (mPFS) of AGC patients who received MT was 10.4 months, and the median overall survival (mOS) was 19.7 months. The author believed that the activity and toxicity characteristics of capecitabine as a maintenance agent for AGC seem to be quite favorable. Gong et al. (2014) used paclitaxel plus capecitabine as the 1L of AGC and continued the MT of capecitabine monotherapy for patients with no disease progression. It was found that the PFS and OS were 188 days and 354 days, respectively. Among the 45 patients who received capecitabine monotherapy after 1L, there was no disease progression, and the OS was significantly prolonged (531 days). The adverse reaction to MT was mild (Gong et al., 2014). Qiu et al. (2014) studied the efficacy and safety of capecitabine as a post-1L MT in AGC patients using oxaliplatin and capecitabine. They found that the mPFS and mOS of MT patients were 11.4 months and 23 months, respectively, while those of the control group without MT were 7.1 months and 14.7 months, respectively. The difference was statistically significant (*p* < 0.001). Multivariate analysis indicated that MT was an independent prognostic factor for patients with AGC (Qiu et al., 2014). Lu et al. (2015) conducted a prospective study on capecitabine as MT after capecitabine-based combination chemotherapy in patients with advanced esophagogastric junction adenocarcinoma. The results showed that the mPFS of patients receiving MT was 11 months and the mOS was 17 months, which were significantly longer than 7 months and 11 months in the control group, respectively (*p* < 0.001) (Lu et al., 2015). Arslan and Atilla (2022) evaluated the efficacy and toxicity of capecitabine MT in MGC patients who received the combination of docetaxel + cisplatin + 5-FU as the 1L treatment. It was found that maintenance with capecitabine might increase the mPFS and mOS.

Multiple studies have investigated the efficacy and safety of S-1 monotherapy as MT for AGC and MGC. Tang et al. (2020) evaluated the efficacy and safety of MT in AGC patients following D2 gastrectomy after treatment with SOX chemotherapy regimens. A total of 122 patients received maintenance chemotherapy for S-1 (MT group), while 133 patients did not receive MT (control group). All cases were followed up, and OS, recurrence-free survival (RFS), and toxicity were compared. It was found that the MT group showed significantly higher 5-year OS (*p* < 0.05) and RFS (*p* < 0.05) than the control group. The incidence of hand-foot syndrome was significantly higher in the MT group (*p* < 0.05). No deaths related to toxicity occurred. It was indicated that S-1 maintenance chemotherapy after SOX regimen chemotherapy provided significant survival benefits for patients with AGC after D2 gastrectomy. Petrioli et al. (2015) administered leucovorin/bolus and continuous infusion of 5-FU as maintenance chemotherapy for elderly patients with advanced esophagogastric cancer who had poor performance status. It was found that the disease control rate within 6 months was 47.3%. The mPFS was 5.9 months, and the mOS was 9.6 months. The author believed that after FOLFOX-4, the administration of leucovorin/bolus and continuous infusion of 5-FU maintenance chemotherapy seemed to be an active and well-tolerated treatment strategy for elderly patients with advanced esophagogastric cancer.

### 3.2 Frontier exploration progress

With the rapid progress in the treatment of malignant tumors, numerous studies have explored various MT modalities, including single-agent targeted drugs or immunosuppressants, combination chemotherapy, and regimens integrating targeted drugs, immunosuppressants, or traditional Chinese medicine (TCM) with chemotherapy, all of which have shown potential value for GC maintenance.

#### 3.2.1 Targeted therapy maintenance strategy

The application of targeted therapy maintenance strategies in GC aims to inhibit tumor growth, spread, and recurrence by specifically acting on the key molecular targets of tumor cells, thereby prolonging the PFS and OS of patients and improving QoL (Luo et al., 2025; Skórzewska et al., 2023). Common targeted drugs, including anti-angiogenic drugs (such as bevacizumab), anti-(human epidermal growth factor receptor-2, HER2) drugs (such as trastuzumab), and inhibitors targeting other signaling pathways, have significant clinical significance in the maintenance stage of GC patients (Guan et al., 2023; Li et al., 2024). Anti-angiogenic drugs such as ramucirumab and bevacizumab have shown significant efficacy in the treatment of GC. Ramucirumab inhibits tumor angiogenesis by blocking VEGFR and has been shown to prolong mOS in second-line treatment (Yuan et al., 2025). Although previous trials showed that dual chemotherapy with oxaliplatin and irinotecan, along with this dual-drug regimen combined with cetuximab, achieved high response rates in AGC, these treatments were associated with a very short time to progression. This suggested a tendency to develop chemoresistance. Wöll et al. (2017) investigated the combination of sequential chemotherapy (oxalate and irinotecan, followed by docetaxel) with bevacizumab in the GASTRIC-3 trial. Patients who have achieved at least stable disease levels continue to take bevacizumab. The objective response rates among the 33 patients were 12.1% for complete response (CR), 39.4% for partial response (PR), and 27.3% for stable disease (SD). The mPFS was 7.0 months, and the mOS was 11 months. It was worth noting that two patients continued to receive bevacizumab MT for more than 5 years and continuously underwent CR. However, one patient still had living tumor residues in the subsequent gastroscopy biopsy, highlighting the fact that bevacizumab monotherapy could control the disease at least, in this patient. This indicates sequential chemotherapy combining oxaliplatin/irinotecan with docetaxel and bevacizumab, followed by maintenance with bevacizumab, is a feasible approach for AGC. For patients with HER2-positive GC, trastuzumab is the preferred targeted therapeutic drug. After the initial treatment, trastuzumab can be used as part of MT to continuously inhibit the HER2 signaling pathway and prevent tumor progression (Guan et al., 2023). Furthermore, novel HER2-targeted drugs such as lapatinib and T-DM1 have also shown potential value for MT in research. For GC patients with positive tropomyosin receptor kinase (TRK) fusion genes, TRK inhibitors larotrectinib and entrectinib are effective targeted therapeutic drugs. These drugs can be used as part of the MT after the initial treatment, continuously inhibiting the TRK signaling pathway and delaying tumor progression (Smyth and Cunningham, 2012). Bramhall et al. (2002) randomly assigned marimastat or placebo to MT in patients with gastric and gastroesophageal adenocarcinoma who received 5-FU chemotherapy as a 1L regimen. It was found that the mOS for the placebo was 138 days, and for marimastat, it was 160 days. The 2-year survival rates were 3% and 9%, respectively. Compared with the placebo, the PFS of patients receiving marimastat was also significantly longer (*p* < 0.05). These results supported the possible role of marimastat as an MT after a chemotherapy response or stabilization of the disease. The Parallel-303 study randomly assigned patients with inoperable locally AGC or MGC who responded to platinum-based 1L chemotherapy in a 1:1 ratio to receive MT with the PARP inhibitor pamiparib (n = 71) or placebo (n = 65). Compared with the placebo, the mPFS with pamiparib maintenance was longer (2.1 months vs. 3.7 months), and it was well tolerated with few treatment discontinuations. No unexpected safety signals were found (Ciardiello et al., 2023). Drugs targeting signaling pathways such as EGFR, MET, and PI3K/AKT/mTOR have also shown potential in clinical trials, although their efficacy still needs further verification (Li et al., 2024). The aim of a double-blind, placebo-controlled, randomized, multicenter phase 3 study conducted in Asia, Australia, Europe, and North America was to compare the efficacy, safety, and tolerability of the PARP inhibitor pamiparib with placebo in MT in 540 patients with AGC (Ciardiello et al., 2018). These patients responded to 1L, platinum-based chemotherapy. The primary endpoint was PFS. The key secondary endpoints included safety/tolerability, OS, objective response rate, response time and duration, and the duration of the second follow-up treatment. Relevant biomarker analyses were conducted on tumor tissues and blood.

#### 3.2.2 Maintenance strategies for immunotherapy

In recent years, emerging therapies have been explored in clinical trials as regimens for MT after 1L treatment in the main GC population. Due to its low toxicity and high efficiency, immunotherapy has become one of the ideal choices for MT. Commonly used drugs include immune checkpoint inhibitors (such as avelumab), which have shown significant efficacy in the treatment of GC. During the MT stage, these drugs can be used alone to continuously activate the patient’s immune system and inhibit tumor growth (Apicella et al., 2017). Avelumab is a human IgG1 anti-PD-L1 monoclonal antibody that has shown persistent clinical activity across a range of tumors. In the phase IB cohort (Chun et al., 2019), 150 patients with advanced or metastatic GC/gastroesophageal junction cancer (GEJC) were included in two subgroups and received avelumab as 1L maintenance or second-line treatment. The results showed that avelumab was well tolerated and demonstrated promising clinical activity. Specifically, in the 1L maintenance subgroup, the overall response rate was 6.7% (complete response was 2.2%), and the mOS was 11.1 months from the start of avelumab treatment and 18.7 months from the start of 1L chemotherapy. Moehler et al. (2020) compared the efficacy of MT with avelumab versus continuous chemotherapy in patients with advanced GC/GEJC after the IL chemotherapy. It was found that avelumab as MT showed significant survival benefits in patients with GC/GEJC. The ORR after randomization was similar in the two groups (13.3% vs. 14.4%), the duration of response was longer in the avelumab group (62.3% vs. 28.4% at 12 months after randomization), and the incidence of TRAEs was lower in the avelumab group (61.3% vs. 77.3%). The grades were 12.8% and 32.8%, respectively.

#### 3.2.3 Combination medication strategy for MT

Combination chemotherapy and regimens that integrate targeted drugs, immunosuppressants, or TCM with chemotherapy have all shown potential value for GC maintenance.

Several studies have focused on the MT of combined chemotherapy regimens in AGC and MGC, involving the comparison of the efficacy, safety, and treatment strategies of regimens such as S-1 or combined with platinum/5-FU. Some studies have shown that MT can bring survival benefits. The MATEO trial is an ongoing open-label, multicenter, randomized phase II study aimed at investigating the efficacy of receiving S-1 monotherapy compared with continuing the same regimen as 1L platinum-5-FU chemotherapy as MT (Haag et al., 2017). The primary endpoint was the OS rate, and the secondary endpoints included safety and toxicity, PFS, and QoL. The result has not been made public yet. Park et al. (2017) compared the efficacy of continuous SOX until disease progression (continuous arm) or no chemotherapy interval after six cycles of 1L chemotherapy with that of the SOX regimen in MGC patients, followed by reintroduction of SOX at progression (stop-and-go arm). The results showed that the PFS of the continuous arm was significantly longer than that of the stop-and-go arm (10.5 months vs. 7.2 months). Walden et al. (2021) evaluated the efficacy and tolerability of maintenance and continuous treatment in advanced GC/GEJC through a retrospective study. Patients who initially received induction therapy with 5-FU and platinum and achieved at least stable disease after 16 weeks were assigned to different groups. Those who continued induction chemotherapy were classified as the continuous group, whereas those who switched to maintenance 5-FU monotherapy or observation were classified as the maintenance group. It was found that there was no significant difference in PFS and OS between the continuous group and the maintenance group. However, the studies by Park and Walden both indicated that the incidence and grade of AEs in the MT and stop-and-go arm were lower than those in the continuous group. This was particularly important because the treatment goal for advanced or metastatic diseases is to prolong life while maintaining the QoL. Therefore, both Park and Walden suggested that patients who can tolerate induction therapy and at least achieve stable disease should not continue with the same regimen as 1L chemotherapy. They may be more suitable to receive single-agent 5-FU MT or reintroduced treatment after observing the onset of the disease.

In a phase III trial (Randon et al., 2024), among patients with advanced HER2-negative GC/GEJC, the mPFS of patients with ramucirumab plus paclitaxel as switch maintenance was 6.6 months. The continuation of 1L oxaliplatin-based chemotherapy (control group) was 3.5 months (*p* < 0.001). In the analysis of the 24-month restricted mean survival time, the restricted mean PFS of the switch maintenance group was 8.8 months, and that of the control group was 6.1 months (*p* = 0.001). Moreover, the incidence of TRAEs in the switch maintenance group was also significantly lower than that in the control group, indicating that for patients with advanced HER2-negative GC/GEJC who did not meet the conditions for immunotherapy or targeted drugs, maintaining the ramucirumab plus paclitaxel switch may be a potential therapeutic strategy. Wang et al. (2024b) reported the efficacy and safety of the combination of chemotherapy and targeted therapy as MT for GC patients through a case report. A 68-year-old man diagnosed with HER2-negative advanced GC complicated with liver metastasis was evaluated as having PR efficacy after receiving six cycles of 1L treatment with oxaliplatin plus S-1 chemotherapy from December 2014 to May 2015. Due to poor physical condition, the patient was in a cachectic state and was unable to receive second-line treatment for GC. After the patient received MT with oral S-1 plus apatinib, the tolerance was good. Regular tumor examinations were conducted, the condition was stable, and the patient survived for more than 7 years (106 months) without progression. Trastuzumab combined with chemotherapy is an effective treatment method for HER2-positive AGC. However, the optimal MT for patients who benefit from 1L treatment remains unclear. Li et al. (2020) conducted a prospective observational study. They divided HER2-positive AGC patients receiving six cycles of trastuzumab-based 1L chemotherapy into two groups based on maintenance strategies: trastuzumab monotherapy (arm A) and trastuzumab plus a mono-chemo-agent derived from the initial chemotherapy (arm B). The results showed no significant difference in mOS (16.5 vs. 20.0 months) or mPFS (7.9 vs. 11.0) between the two groups. However, adding a chemotherapeutic agent reduced the risk of death by 29%. The author believed that the combination of single chemical agents and trastuzumab, in addition to the incremental cost-effectiveness ratios of PFS, for patients who benefited from trastuzumab-based 1L therapy in the first six cycles, especially those with certain clinical or treatment-related characteristics, showed advantages in the absolute value and hazard ratio in OS. A large number of random trials with samples are needed. Bergen et al. (2023) evaluated the efficacy of trastuzumab (T) plus platinum salt + 5-FU (F) and T alone as maintenance regimens after 1L chemotherapy (F + T) for advanced HER2-positive esophago-gastric adenocarcinoma. It was found that adding F to T monotherapy as MT could prolong the PFS and OS of nursing patients. Reintroducing the initial treatment at the first progression could significantly prolong OS, which might be a feasible method to retain the later treatment line.

It was well known that based on CHECKMATE649 and REORIENT 16, the combination of chemotherapy and PD-1 immunotherapy had become the standard 1L treatment for AGC. The optimal MT strategy after 1L chemotherapy combined with PD-1 immunotherapy remains unclear. Chen et al. (2024) divided 84 patients with AGC or MGC who did not experience disease progression after 1L treatment with oxaliplatin-based chemotherapy plus a PD-1 antibody into two groups: those who received capecitabine plus a PD-1 antibody (sintilimab, tislelizumab, or nivolumab) (n = 44) and those who received capecitabine monotherapy (n = 40) as MT. It was found that the mPFS (8.4 months vs. 7.3 months, *p* = 0.005) and mOS (16.8 months vs. 14.6 months, *p* = 0.02) of patients in the capecitabine plus PD-1 antibody group were significantly longer than those in the capecitabine monotherapy group. There was no significant difference in TRAEs between the capecitabine plus PD-1 antibody group and the capecitabine group. Most TRAEs were tolerable. The addition of PD-1 antibodies did not significantly increase the incidence of adverse reactions.

Kong et al. (2024) conducted a study aimed at evaluating the efficacy and safety of the “Fuzheng jiedu Quyu Method” (FJQR) combined with 5-FU as MT for patients with HER-2-negative GC. Among the 129 eligible patients, 64 were assigned to the treatment group receiving FJQR plus 5-FU, while 65 were assigned to the control group receiving 5-FU alone. It was found that compared with the control group, the mPFS of the treatment group was significantly prolonged (6.3 months vs. 5.0 months, *p* = 0.03). In terms of safety, treatment-related TRAEs were relatively mild in the treatment group, and the incidence of grade III–IV TRAEs could be significantly reduced. This indicated that FJQR plus 5-FU had a synergistic effect in MT in HER2-negative GC and exhibited good efficacy and safety. FJQR can reduce toxicity and improve the efficiency of 5-FU. Hong et al. (2020) aimed to conduct a clinical study on the combination of Xiang Sha Liu Junzi decoction (XSLJZD) and S-1 as MT for stage III or IV GC and colorectal cancer (CRC). Patients with stage III or IV GC and CRC were randomly (1:1) assigned to the S-1 group and the S-1 combined with XSLJZD group for 5 years of MT. The primary endpoint was PFS, and the secondary endpoints were OS and QoL, which included improvements in symptoms before and after treatment, Karnofsky performance status, and AE assessment (Hong et al., 2020).

### 3.3 Specific drugs without demonstrated benefit

Some chemotherapy drugs and PD-L1 inhibitors have not shown significant advantages in the MT of GC. Li et al. (2017) conducted a phase II study to evaluate the efficacy and safety of uracil and tegafur (UFT) in maintaining disease stability or achieving a better response in MGC patients after 1L 5-FU, as assessed by PFS, OS, and safety. However, after the end of this trial, it was not observed that UFT as MT could significantly improve the PFS and OS of patients, and it caused TRAEs such as grade 3–4 anemia, thrombocytopenia, and diarrhea. Fong et al. (2024) randomly assigned 205 patients with HER2-negative advanced esophageal and gastric adenocarcinoma to receive either monitoring (n = 100) or durvalumab MT (n = 105) after 1L chemotherapy. There was no significant difference in PFS and OS between monitoring and bevacizumab. Five patients randomly receiving durvalumab showed an increasing radiological response, while those under monitoring did not. TRAEs occurred in 77 cases (76.2%) of patients assigned to durvalumab, indicating that maintaining durvalumab did not improve the PFS of patients with gastric adenocarcinoma who respond to 1L chemotherapy (Fong et al., 2024). Moehler et al. (2021) investigated the effect of avelumab MT after 1L induction chemotherapy for GC/GEJC. A total of 805 patients received induction, and 499 were randomly assigned to avelumab (n = 249) or continued chemotherapy (n = 250). It was found that the mOS rates of avelumab and chemotherapy were 10.4 months and 10.9 months, respectively, and the 24-month OS rates were 22.1% and 15.5%, respectively. In patients with overall advanced GC or GEJC or in the pre-specified PD-L1 positive population, JAVELIN Gastric 100 did not exhibit superior OS with avelumab maintenance compared with continuous chemotherapy (Table 1).

**TABLE 1:** Efficacy and safety outcomes in agents used in clinical trials of maintenance therapy.

Study	Study design	Number of patients	Study population	Drug	Efficacy	Safety outcome	Trial status
Current status of guideline recommendations							
Eren et al. (2016)	Retrospective Study	11	AGC	Capecitabine	The mPFS was 10.4 months, and the mOS was 19.7 months	No death or hospitalization for capecitabine toxicity was noted. One patient developed grade III HFS, and one patient developed grade I HFS. Grade II diarrhea and mucositis was developed in one patient. Mild hematologic toxicity and grade II anemia were detected in one patient	Completed
Gong et al. (2014)	Phase II	194	AGC	Capecitabine	The objective response rate was 34.8%, and the PFS and OS were 188 days and 354 days, respectively	TRAEs were mild. The most common grade 3–4 toxicities were leukopenia and neutropenia	Completed
Qiu et al. (2014)	Prospective observation	64 Arm A	AGC	Capecitabine	The mPFS was 11.4 months, and the mOS was 23.0 months	TRAEs were mild and controllable	Completed
Lu et al. (2015)	Randomized	30 Arm A	Advanced esophagogastric junction adenocarcinoma	Capecitabine	The mPFS was 11.0 months, and the mOS was 27.0 months	TRAEs were tolerable	Completed
Arslan and Atilla (2022)	Retrospective Study	10	MGC	Capecitabine	The mPFS and mOS were increased.	Not reported	Completed
Tang et al. (2020)	Phase III	122 MCT group	GC	S-1	The 5-year OS and recurrence-free survival were significantly higher than those of the control group patients who did not receive MT.	The incidence of HFS was significantly higher, and no deaths related to toxicity occurred	Completed
Petrioli et al. (2015)	Not reported	32	Advanced esophagogastric cancer	Leucovorin/bolus and continuous infusion 5-FU	The disease control rate at 6 months was 47.3%, the mPFS was 5.9 months, and the mOS was 9.6 months	Six patients presented with grade 3 neutropenia (15.7%), and two patients presented with grade 3 anemia and thrombocytopenia (5.2%)	Completed
Frontier exploration progress							
Targeted therapy maintenance strategy							
Wöll et al. (2017)	GASTRIC-3 trial	33	Inoperable locally advanced or metastatic gastric cancer	Bevacizumab	The objective response rates were CR 12.1%, PR 39.4%, and SD 27.3%. The mPFS was 7.0 months, and the mOS was 11 months	Not reported	Completed
Bramhall et al. (2002)	Randomized trial	185	AGC	Marimastat	The PFS was significantly longer, the mOS was 160 days, and the 2-year survival rate was 9%	Marimastat treatment was associated with the development of musculoskeletal pain and inflammation	Completed
Ciardiello et al. (2023)	Phase II	71	AGC	Pamiparib	The mPFS was 3.7 months, and the mOS was 10.2 months	TRAEs had good tolerance, and treatment was rarely discontinued. No unexpected safety signals were found	​
Ciardiello et al. (2018)	Phase III	270	Inoperable locally advanced or metastatic gastric cancer	Pamiparib	The primary endpoint was PFS. The key secondary endpoints included safety/tolerability, OS rate, objective response rate, response time and duration, and the duration of the second follow-up treatment	Not reported	Ongoing
Maintenance strategies for immunotherapy							
Moehler et al. (2020)	Phase III	249	HER2- advanced GC or gastroesophageal junction cancer (GEJC)	Avelumab	The reaction lasted longer	The incidence of TRAEs was relatively low	Completed
Chun et al. (2019)	Phase IB	90	Advanced GC/GEJC	Avelumab	The overall response rate was 6.7% (complete response was 2.2%), mOS was 11.1 months after the start of avelumab treatment and 18.7 months after the start of 1L chemotherapy, and mPFS was 2.8 months	Other common TRAEs of any grade include fatigue (10.0%) and nausea (6.7%). Serious AEs related to treatment occurred in 4.0% of the patients	Completed
The combination medication strategy for MT							
Haag et al. (2017)	Phase II	Not reported	Her-2-negative esophago-gastric adenocarcinoma	S-1 alone or continued platinum-fluoropyrimidine-based chemotherapy	The primary endpoint was OS, and the secondary endpoints included PFS and QoL	Secondary endpoints included safety and toxicity	Ongoing
Park et al. (2017)	Phase II	59 continuous arm; 62 stop-and-go arm	MGC	SOX	The PFS of the continuous arm was significantly longer than that of the stop-and-go arm (10.5 months vs. 7.2 months)	The continuous arm reported a significantly higher incidence of TRAEs	Completed
Walden et al. (2021)	Retrospective analysis	48 continuous group; 42 maintenance/observation group	Advanced gastric and gastroesophageal junction	5-FU with platinum-based chemotherapy (continuous group); 5-FU (maintenance/observation group)	There was no significant difference in PFS and OS between the two groups	The continuous arm reported a significantly higher incidence of TRAEs	Completed
Randon et al. (2024)	Phase III	144	HER2-negative GC/GEJC	Ramucirumab plus paclitaxel	The mPFS was 6.6 months. In the analysis of the 24-month restricted mean survival time, the restricted mean PFS was 8.8 months	The incidence of TRAEs had decreased significantly	Completed
Wang et al. (2024b)	Case report	1	AGC with liver metastasis	S-1 plus apatinib	The condition was stable. The patient had survived for more than 7 years (106 months) without any progression	Not reported	Completed
Li et al. (2020)	Prospective observational study	48	HER2-positive AGC	trastuzumab plus mono-chemo-agent	The mPFS was 11.0 months, and the mOS was 20.0 months	It can reduce the risk of death by 29%	Completed
Bergen et al. (2023)	Retrospective study	86	Advanced HER2-positive esophago-gastric adenocarcinoma	Trastuzumab plus platinum salt plus 5-FU	Prolonged the PFS and OS of patients in care	Not reported	Completed
Chen et al. (2024)	Pilot analysis	44	Advanced or metastatic GC	Capecitabine plus PD-1 antibodies (sintilimab, tislelizumab, or nivolumab)	The mPFS was 8.4 months, and the mOS was 16.8 months	Most TRAEs were tolerable. There was no significant increase in TRAEs	Completed
Kong et al. (2024)	Randomized study	64	HER-2-negative gastric cancer	Fuzheng jiedu Quyu method combined with 5-FU	The mPFS was significantly prolonged (6.3 months)	TRAEs were relatively mild, and the incidence of grade III–IV TRAEs was significantly reduced	Completed
Hong et al. (2020)	Randomized study	Not reported	Stage III or IV gastric carcinoma	Xiang Sha Liu Junzi decoction plus S-1	The primary endpoint was PFS, and the secondary endpoints were OS and QoL, which included the improvement of symptoms before and after treatment, along with the performance status of Karnofsky	The secondary endpoint was the assessment of TRAEs	Ongoing
Drugs without demonstrated benefit							
Li et al. (2017)	Phase II	58	MGC	Uracil plus tegafur	It cannot significantly improve the PFS and OS of the patients	It caused grade 3–4 anemia, thrombocytopenia, and diarrhea	Completed
Fong et al. (2024)	Randomized study	105	HER2-negative advanced esophago-gastric adenocarcinoma	Durvalumab	There was no improvement in PFS	Among the 77 cases (76.2%) of patients, TRAEs occurred	Completed
Moehler et al. (2021)	Phase III	249	GC	Avelumab and chemotherapy	It did not demonstrate an excellent OS	TRAEs occurred in 149 patients (61.3%), including 31 patients (12.8%) with grade ≥3 TRAEs	Completed

The SOX regimen was S-1 plus oxaliplatin.

HFS, hand foot syndrome; GC, gastric cancer; AGC, advanced gastric cancer; MGC, metastatic gastric cancer; CR, complete response; PR, partial response; SD, stable disease; TRAEs, treatment-related adverse events; AEs, adverse events; QoL, quality of life; PFS, progression-free survival; OS, overall survival; mPFS, median progression-free survival; mOS, median overall survival; MT, maintenance therapy; 5-FU, 5-fluorouracil.

## 4 Exploration of potential biomarkers for MT of GC

**FIGURE 2:**
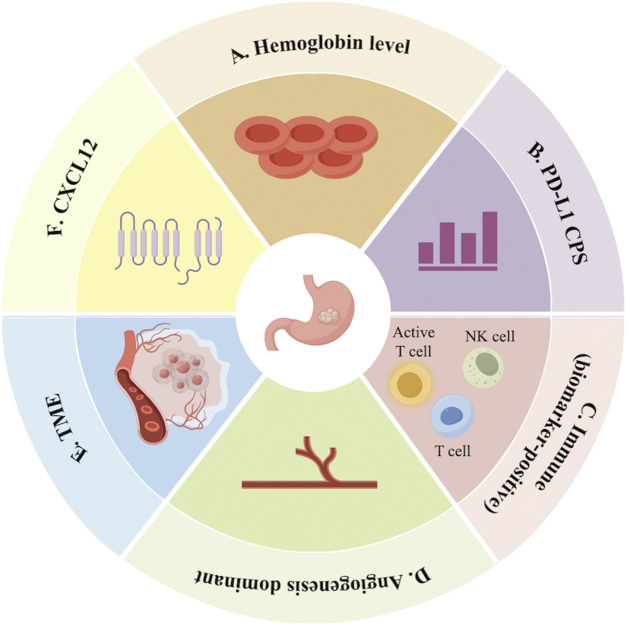
Exploration of potential biomarkers for MT of GC (A: hemoglobin level; B: PD-L1 CPS; C: immune [biomarker-positive]; D: angiogenesis-dominant; E: TME; F: CXCL12).

Exploring biomarkers related to MT for GC holds significant clinical importance for guiding treatment plan selection, predicting treatment response, evaluating prognosis, and optimizing individualized treatment. It helps reduce ineffective treatment, minimize adverse reactions, enhance survival benefits, and provide a target direction for drug development. Currently identified biomarkers can be classified, based on detection methods, into those derived from liquid biopsy and those based on pathological tissues (Figure 2).

### 4.1 Liquid biopsy-based biomarkers

Biomarkers based on liquid biopsy include hemoglobin (HGB) and C-X-C chemokine ligand 12 (CXCL12). HGB levels have previously been identified as predictive and prognostic factors for AGC, and patients with low-baseline HGB levels rarely benefit from second-line chemotherapy (Ji et al., 2009). When Li et al. (2017) evaluated the efficacy and safety of UFT as MT in patients with MGC following 1L 5-FU-based chemotherapy, they found that low-baseline HGB (<120 g/L) was associated with poor PFS in MT (*p* = 0.032), while patients with normal HGB levels benefited from UFT treatment (*p* = 0.008). Therefore, this study held that the normal HGB level at baseline was a predictive biomarker for patients to have better outcomes during MT. Park et al. (2006) also found that in AGC patients who received 1L chemotherapy based on 5-FU, anemia was closely associated with reduced response rate, PFS, and OS, and there was a significant correlation between normal baseline HGB levels as predictive biomarkers for MT strategies. Nearly half of the patients in this study had normal HGB levels at baseline. When these patients received UFT treatment, their PFS increased significantly (by 4.7 months, with a *p*-value of 0.032), and in the OS analysis, the survival curves also separated, with an increase of 13 months. In contrast, patients with low HGB levels (80–120 g per liter) had poorer PFS and showed no improvement in the survival rate. CXCL12 is involved in the regulation of tumor progression and the TME (Suarez-Carmona et al., 2021). Research has found that CXCL12 is a downstream target of circDLG1, which may promote the infiltration of MDSCs into the TME to mediate resistance to anti-PD-1 in GC (Chen et al., 2021). Studies have shown that CXCL12 was significantly correlated with the therapeutic effect of PD-1 antibodies in GC (Bouferraa et al., 2022). Chen et al. (2024) investigated the role of CXCL12 in MT for AGC or MGC. Interestingly, in patients with negative CXCL12 expression, the mPFS was 11.5 months in the capecitabine plus PD-1 antibody group and 6.7 months in the capecitabine monotherapy group. In patients with positive expression of CXCL12, the PFS rates of the capecitabine plus PD-1 antibody group and the capecitabine monotherapy group were 9.1 months and 8.9 months, respectively. Similarly, in patients with negative CXCL12 expression, the mOS was 17.7 months in the capecitabine plus PD-1 antibody group and 14.6 months in the capecitabine monotherapy group. Among patients with positive CXCL12 expression, the mOS was 17.6 months in the capecitabine plus PD-1 antibody group and 16.7 months in the capecitabine monotherapy group. Therefore, this study further indicated that in patients receiving PD-1 MT, the expression of CXCL12 was inversely proportional to PFS and OS. To our knowledge, this was the first study to explore biomarkers that predict the prognosis of GC patients receiving PD-1-based MT.

### 4.2 Pathological tissue-based biomarkers

Biomarkers based on pathological tissues include PD-L1 combined positive score (CPS), immune (biomarker-positive), and angiogenesis-dominant (biomarker-negative) biomarkers. Fong et al. (2024) conducted an exploratory survival analysis in a study evaluating MT for advanced esophago-gastric adenocarcinoma, based on CPS, immune (biomarker-positive) or angiogenesis dominant (biomarker-negative) biomarkers, and TME phenotypes. It was found that, compared with the PD-L1 CPS<5 and angiogenesis-dominant (biomarker-negative) subgroups, respectively, MT was beneficial for the monitoring effect of OS in patients with CPS ≥5 and immune (biomarker-positive) subgroups. The exploratory analysis by Moehler et al. (2021), using the Xerna TME RNA panel, indicated that durvalumab might be beneficial for biomarker-positive GC patients and may expand the options for immunotreatment-sensitive patients, even in populations with PD-L1 CPS ≥ 5. Biomarkers such as HRD status (PARPi) and PD-L1 expression may improve treatment options. TME characteristic analysis can further optimize the patient selection for anti-PD-L1 treatment based solely on PD-L1 CPS. Chen et al. (2024) also found that in GC patients receiving PD-1 MT, PD-L1 expression was significantly correlated with PFS and OS. In patients with high PD-L1 expression (CPS ≥5), the median PFS was 12.2 months in the capecitabine plus PD-1 antibody group and 8.7 months in the capecitabine monotherapy group (*p* < 0.0001). In patients with low PD-L1 expression (CPS<5), the median PFS was 8.4 months in the capecitabine plus PD-1 antibody group and 7.3 months in the capecitabine monotherapy group (*p* = 0.005). Similarly, in patients with high PD-L1 expression (CPS ≥5), OS was significantly improved (18.6 vs. 16.3 months, *p* = 0.005), while in patients with low PD-L1 expression (CPS<5), the median OS rates of the capecitabine plus PD-1 antibody group and the capecitabine group were 16.8 months and 14.6 months, respectively (*p* = 0.02).

## 5 Discussion and conclusion

This article systematically reviewed the studies on MT for GC, indicating that there was still interest in evaluating this treatment approach. MT plays a significant role in the management of GC. Whether as a continuous or switching strategy, its core objective is to control disease progression, prolong PFS and OS, and improve the QoL of patients through continuous drug intervention. With the development of immunotherapy and targeted therapy, the strategies for MT of GC are constantly being optimized, providing patients with more treatment options and hope.

Although significant progress has been made in MT for GC, there are still limitations in terms of heterogeneity of evidence, patient selection, and biomarkers. Current evidence shows certain heterogeneity in clinical trials. Moehler et al. (2020) found that avelumab, as MT, showed significant survival benefits in GC/GEJC patients, but the JAVELIN Gastric 100 study failed to demonstrate the superiority of avelumab as MT (Moehler et al., 2021). This indicates that different trial designs and inclusion criteria may mask consistent therapeutic benefits. Patient selection remains challenging, and individual patient characteristics are complex. Therefore, clinically similar cohorts may have different molecular characteristics, thereby affecting treatment response. However, the lack of standardized and comprehensive biomarkers makes the situation even more complicated. Future research can achieve this by conducting standardized, multi-center, randomized controlled trials that uniformly define the intervention plan, inclusion criteria, and efficacy evaluation system to reduce evidence heterogeneity; combine clinical characteristics and molecular typing to conduct precise stratified studies, build individualized prediction models, and optimize patient selection to enhance the targeted nature and benefit rate of MT; and further carry out validation studies on emerging biomarkers based on genomics, transcriptomics, metabolomics, imagingomics, and pathological omics to promote the development of GC MT toward a more precise and individualized direction (Yan et al., 2023; Wang et al., 2024c).

## Glossary

1L: first-line
5-FU: 5-fluorouracil
AEs: adverse events
AGC: advanced gastric cancer
CEP: circulating endothelial precursor
CR: complete response
GEJC: gastroesophageal junction cancer
GC: gastric cancer
CPS: combined positive score
CRC: colorectal cancer
CTX: cyclophosphamide
CXCL12: C-X-C motif chemokine ligand 12
DCs: dendritic cells
DTCs: dormant tumor cells
FJQR: Fuzheng jiedu Quyu method
HER2: human epidermal growth factor receptor-2
HGB: hemoglobin
LDM: low-dose chemotherapy
MDSCs: myeloid-derived suppressor cells
MGC: metastatic gastric cancer
mPFS: median progression-free survival
mOS: median overall survival
MT: maintenance therapy
MTD: maximum tolerated dose of chemotherapy
NK: natural killer
OS: overall survival
PD-1: programmed death-1
PD-L1: programmed death-ligand 1
PFS: progression-free survival
PR: partial response
PTX: paclitaxel
QoL: quality of life
RFS: recurrence-free survival
SD: stable disease
SOX: S-1 and oxaliplatin
TCM: traditional Chinese medicine
TME: tumor microenvironment
TRAEs: treatment-related adverse events
Tregs: regulatory T cells
UFT: uracil and tegafur
VEGF: vascular endothelial growth factor
VEGFR: vascular endothelial growth factor receptor
XSLJZD: Xiang Sha Liu Junzi decoction
